# Relationship between the structure and microcirculation of the optic disc region and myopic traction maculopathy in highly myopic eyes

**DOI:** 10.1007/s00417-023-06312-w

**Published:** 2023-11-13

**Authors:** Yang Bai, Jinyuan Sui, Haoru Li, Qing He, Ruihua Wei

**Affiliations:** 1 Tianjin Key Laboratory of Retinal Functions and Diseases, Tianjin Branch of National Clinical Research Center for Ocular Disease, Eye Institute and School of Optometry, Tianjin Medical University Eye Hospital, Tianjin, China; 2 Tianjin Binhai High-Tech Industrial Development Zone, No. 251 Fukang Road, Huayuan Industrial Zone (Nankai District), Tianjin, China

**Keywords:** High myopia, Myopic traction maculopathy, OCTA, Optic disc region structures, Microcirculation

## Abstract

**Purpose:**

To explore the characteristics and influencing factors structural and microcirculatory of optic disc and peripapillary tissue in eyes with myopia traction maculopathy (MTM).

**Methods:**

There were 100 eyes from 77 patients in this study. We used 1:1 matching axial length in myopic eyes. Patients were divided into two groups according to the presence or absence of MTM. Fundus structure parameters were obtained by swept source optical coherence tomography (SS-OCT), and the optic disc microcirculation parameters were obtained by OCT angiography (OCTA).

**Results:**

MTM group were older (*P* = 0.001) and had poorer Best-corrected Visual Acuity (BCVA) (*P* = 0.011), the optic disc-fovea distance (DFD) was longer (*P* < 0.019), optic disc tilt was greater (*P* < 0.001), area of peripapillary atrophy (PPA) was larger (*P* < 0.001), and PPA/optical disc area (ONH) was higher (*P* < 0.001). The peripapillary scleral thickness (PST) was lower in the MTM group (*P* < 0.001). The mean peripapillary choroidal thickness (PCT) (*P* < 0.001) and PCT in the 10 orientations were significantly lower in the MTM group than in the NMTM group (all *P* < 0.01). Vascular density in the nasosuperior (NS) region of the optic disc was significantly lower in the MTM group (*P* = 0.037). The generalized estimating equation suggested that PPA area (*P* = 0.028), mean PCT (*P* = 0.008), superior PCT (*P* = 0.027), inferonasal PCT (*P* = 0.040), temporoinferior PCT (*P* = 0.013), and PST (*P* = 0.046) correlated with MTM. Age, axial length, optic disc tilt, PPA area, mean PCT, and optic disc central zone (0–2 mm) vascular density (all *P* < 0.05) were significantly correlated with PST.

**Conclusions:**

The enlarged PPA area and thinner PCT and PST in eyes with MTM are more significant. Lower PST in high myopia was related to abnormalities of PCT and microcirculation.

**Trial registration:**

Clinical Trial Registration number: ChiCTR2100046590


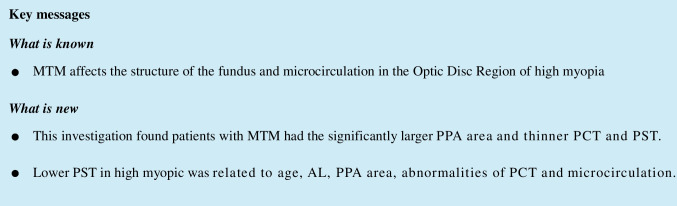


## Introduction

The prevalence of high myopia is increasing every year and has become a major public health problem and is expected to be one of the leading reasons of global visual damage in the coming decades. By 2050, visual damage due to high myopic fundus degeneration is projected to increase to 55.7 million. The prevalence of pathologic myopia in Asian populations ranges from 12 to 27% [1]. Myopic traction maculopathy (MTM) is the leading cause of vision loss, particularly in the East Asian population [2]. An increase in the axial length (AL), the presence of posterior staphyloma (PS), and atrophy of the retinal choroid are considered the main risk factors for MTM [3–5].

It has been shown that morphological changes in the optic disc region, such as tilt, rotation, and peripapillary atrophy (PPA), may indicate the progression of high myopia [6, 7]. Eyes with MTM showed an increase in the PPA area and changes in the morphology of the optic disc [8–10]. Narrow macular staphyloma confined to the macular area is one of the most important risk factors for MTM [11]. In previous studies on optic disc morphology and MTM, most researchers did not define the type of PS, and the staphyloma located in the optic disc area affected the morphology, resulting in biased results. Microcirculation of the optic disc region is complicated, mainly through the retinal circulation and peripapillary choroidal circulation [12]. Excluding the influence of PS on the optic disc region, it is unclear whether MTM affects the structure and microcirculation in the optic disc region. Advances in optical coherence tomography (OCT) have provided technical support. Ultra-wide field (UWF)-SS OCT/OCTA is consistent with 3D MRI in detecting PS [13]. In the eyes of patients with pathologic myopia, improved penetration of deeper tissues allows the shape and thickness of the sclera to be traced and measured, respectively. [14].

Therefore, all PS types of high myopia included in this study were narrow macular staphylomas, and we matched axial lengths of 1:1 to investigate the relationship between the optic disc region structure, microcirculation, and MTM. We selected patients with narrow macular staphyloma reducing biased results. In addition, we analysed the correlations between peripapillary choroidal thickness (PCT), peripapillary scleral thickness (PST), and MTM to provide a basis for analysing the pathological mechanisms of MTM.

## Subjects and methods

### Subjects

This case–control study covered patients with high myopia who visited the Tianjin Medical University Eye Hospital between December 2020 and November 2022. All patients signed an informed consent form and underwent eye examinations. All study procedures conformed to the tenets of the Declaration of Helsinki and were approved by the Ethics Committee of Tianjin Medical University Eye Hospital. The study was registered in the Chinese clinical trial registry (http://www.chictr.org.cn/, Registration number: ChiCTR2100046590). The inclusion criteria were age > 18 years, spherical equivalent refraction (SER) <  − 6.00 D, AL ≥ 26.5 mm, and the type of PS being narrow macular staphyloma. Patients with intraocular pressure (IOP) > 21 mmHg, previous vitreoretinal surgery, primary or secondary glaucoma, systemic disease, refractive interstitial opacity affecting fundus imaging, and poor OCT and OCTA imaging quality were excluded.

### Ophthalmic examinations

Standardized ophthalmic examination given to all patients, including IOP measurement (CT-1; Topcon, Japan), color fundus examination (CR-2, Canon, Japan), slit-lamp biomicroscopy, AL (Lenstar LS-900; Haag-Streit AG, Switzerland) and Autorefraction (KR-800, Topcon, Japan). SS-OCT/OCTA (VG200S; SVision Imaging, Henan, China).

### Morphological characteristic measurement

**Fig. 1:**
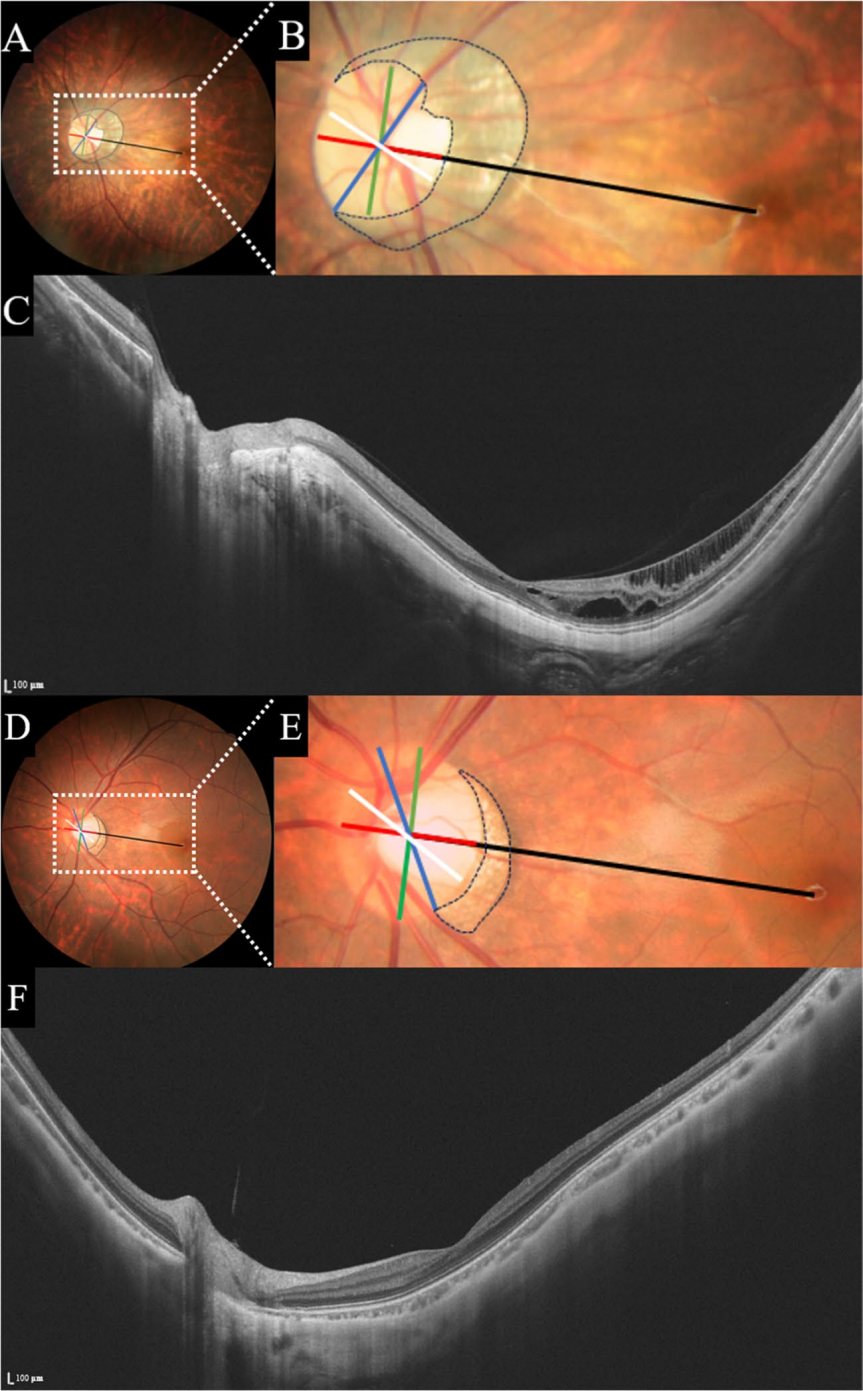
Fundus photography and SS-OCT images of the MTM and NMTM groups. **A–C** Left eye images of a 43-year-old male with MTM, whose AL was 30.27 mm. **D–F** Left eye images of a 34-year-old female with MTM, whose AL was 28.96 mm. **B**,** E** Fundus photograph of a highly myopic eye with the horizontal (red line), vertical (green line), minimal (white line), maximal diameter (blue line), and the PPA (area surrounded by grey lines)

We measured the horizontal, vertical, minimal, and maximal diameter of the optic disc, distance between the optic disc centre and the fovea (“disc–fovea distance,” DFD), optic disc area, and PPA area by using the ImageJ system (National Institutes of Health, Bethesda, MD, USA) in fundus photographs. We calculated optic disc tilt ratio and optic disc rotation degree. The optic disc tilt ratio was measured as the ratio of the maximum and minimum diameters of the optic disc, and the optic discs were considered to be tilted when the tilt ratio exceeded 1.30 [15]. The optic disc rotation was defined as the angle between the long axis of the optic disc and the vertical meridian, and angle greater than 15° was considered to be a rotated optic disc [10]. The PPA is the area of choroidal retinal atrophy where the scleral and choroidal vessels are visible (Fig. 1). Using the Littmann–Bennett method, we corrected for bias in the measurements because of image magnification. All measurements were taken three times by two independent measurers and averaged.

### SS-OCT/OCTA image acquisition

SS-OCT/OCT can analyse an area of 16*16 mm at the centre of the optic disc and a depth of 6 mm. Cube scans measuring 6 mm × 6 mm were centred on the optic disc. Simultaneously, the scan size was adjusted according to the different ALs to avoid differences in magnification. PCT was defined as the vertical distance from the outer edge of the hyperreflective line of the retinal pigment epithelium to the reflective line of the inner sclera at the centre of the optic disc. Scanning the optic disc region automatically obtains 10 clock orientations, superior(S), inferior (I), nasosuperior (NS), nasoinferior (NI), inferonasal (IN), inferotemoporal (IT), temporoinferior (TI), temporosuperior (TS), superotemporal (ST), and superonasal (SN).

**Fig. 2:**
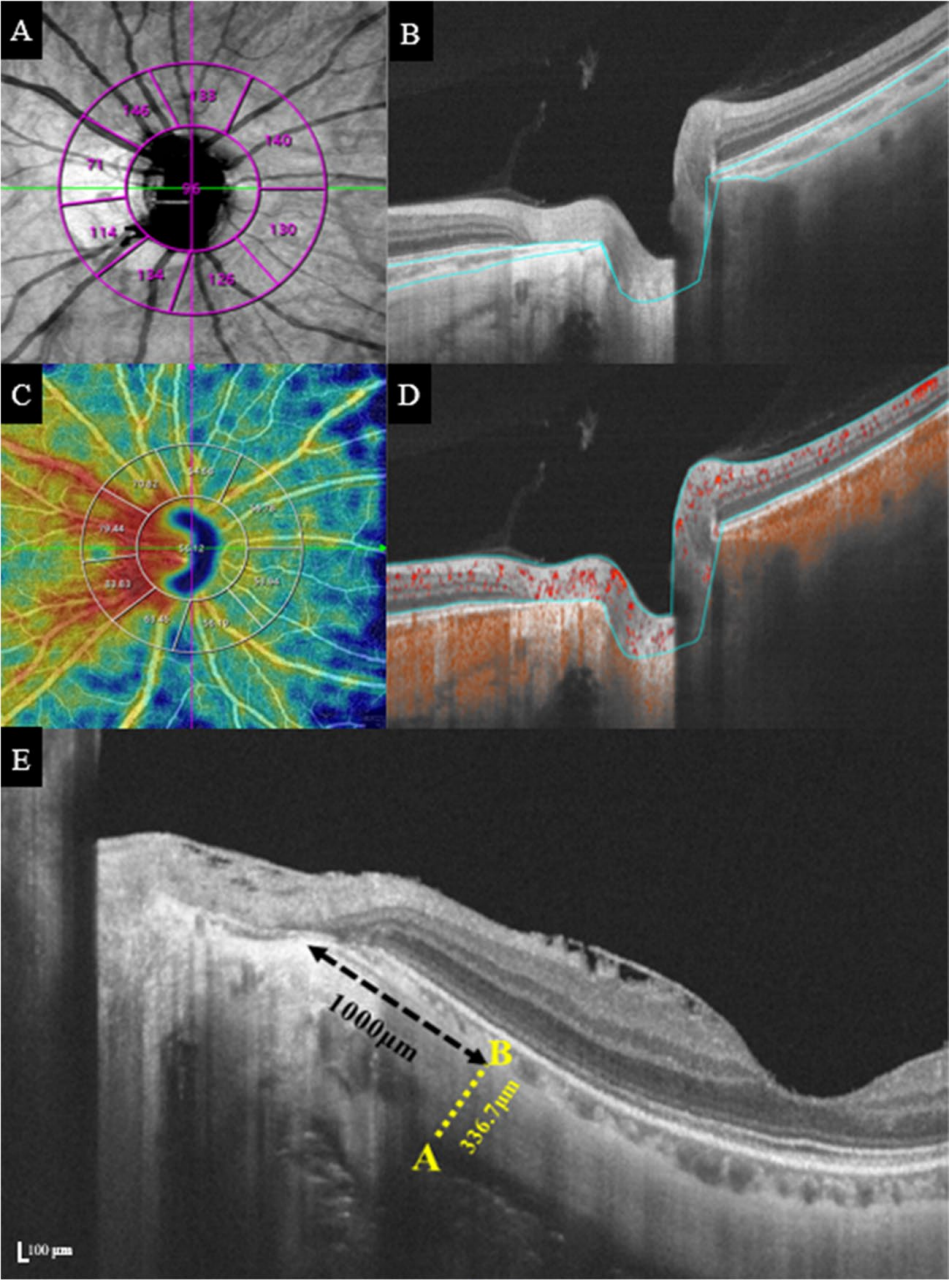
Diagram of microcirculation parameters. **A**,** B** The peripapillary choroidal thickness is automatically identified by the system and defined as the vertical distance between the outer edge of the retinal pigment epithelium (RPE) and the outer edge of the choroid. **C**,** D** The vascular density of Optic disc and peri-optic disc region are automatically identified and calculated by the system. The range of measurement is defined as from the internal limiting membrane to Bruch’s membrane. **E** Schematic diagram of PST measurement

The system automatically identifies and records the PST. PST was measured manually using SS-OCT. The retina was scanned horizontally at 0° in a single-line scan pattern of 16 mm (centred on the optic disc), and the scleral thickness was measured manually at a distance of 1000 μm from the temporal scleral canal on the acquired OCT image. The scleral thickness was measured three times by two experienced ophthalmologists to obtain the average. We analysed vascular density in the optic disc from the internal limiting membrane to the retinal nerve fibre layer, the optic disc region within 2 mm in diameter, and the peri-optic disc region between 2 and 4 mm in diameter. The peri-optic disc region (2–4-mm range) was unevenly divided into eight zones according to the Garway-Heath division of the optic disc: nasosuperior (NS), nasoinferior (NI), inferonasal (IN), inferotemoporal (IT), temporoinferior (TI), temporosuperior (TS), superotemporal (ST), and superonasal (SN) [16], measured vascular density within the optic disc and peri-optic disc region (Fig. 2D).

### Confirmation of the PS and MTM

The presence and location of PS were confirmed using SS-OCT. The diagnosis of PS was the outpouching of the eye wall with a radius of curvature less than that of the surrounding eye wall [17]. The location of the PS was determined according to the Ohno–Matsui classification [18]. Using SS-OCT, all patients were divided into two groups, namely the MTM group and the NMTM group. In the MTM group, based on the MSS staging system, MTM was divided into stage 1, inner/outer maculoschisis, and stage 2, predominantly outer maculoschisis [19]. PS and MTM were observed by two independent measurers. When results were inconsistent, the retinal specialist made the judgement.

### Statistical analysis

Statistical analyses were performed with SPSS software version 26.0 (IBM-SPSS, Inc, Chicago, IL). Continuous variables are expressed as mean ± standard deviation. We compared the groups of eyes with or without MTM by the measured parameters, using the chi-square test, independent *t*-test, and Mann–Whitney *U* test. Due to the fact that the generalized estimating equation (GEE) can eliminate the correlation between left and right eye, we used it to explore the factors influencing the presence of MTM. In the GEE, we used MTM as the independent variable and structure and microcirculation of the optic disc region as the dependent variables. Further, we used linear correlation analysis to analyse the correlation between the PST as the dependent variable and the ocular parameters as the independent variables in highly myopic eyes. *P* < 0.05 was considered a statistically significant difference.

## Results

### Baseline measurements

A total of 100 eyes of 77 patients with high myopia were studied. There were 50 eyes each in the MTM and NMTM groups. A total of 25 eyes had MTM stage 1, and 25 eyes had MTM stage 2. The mean age of the all patients was 42.80 ± 13.41 years old, the mean best-corrected visual acuity (BCVA) (logMAR) was 0.15 ± 0.22, the mean SER was − 14.15 ± 4.35 D, and the mean AL was 29.11 ± 1.24 mm. Patients with MTM were older (*P* = 0.001) and had poorer BCVA (*P* = 0.011), and females were more likely to develop MTM (*P* = 0.007). There was no statistically significant difference between AL (*P* = 0.68) and SER (*P* = 0.88). Compared to MTM Stage 1, those with MTM Stage 2 were older (*P* = 0.09), BCVA was worse (*P* = 0.024), and SER was greater (*P* = 0.04); AL (*P* = 0.38) and sex (*P* = 0.185) have no significant difference in two groups (Table 1).

**Table 1:** Demographics and baseline characteristics of the NMTM and MTM groups

Parameters	NMTM group	MTM group			P1	P2
	(*n* = 50)	Total (*n* = 50)	MTM Stage 1 (*n* = 25)	MTM Stage 2 (*n* = 25)		
Age (y)	38.08 ± 13.00	47.52 ± 12.19	44.60 ± 14.31	50.44 ± 8.98	**0.001**^**‡**^	**0.09**^**‡**^
Gender (male/female)	25/25	12/38	8/17	4/21	0.007^*^	0.185^*^
Iop (mmHg)	16.00 ± 2.90	15.44 ± 2.23	15.27 ± 2.37	15.62 ± 2.11	0.588^**‡**^	0.521^**‡**^
BCVA (logMAR)	0.10 ± 0.18	0.20 ± 0.25	0.11 ± 0.15	0.29 ± 0.30	**0.011**^**‡**^	**0.024**^**‡**^
SER (D)	− 14.09 ± 4.52	− 14.22 ± 4.22	− 13.00 ± 3.50	− 15.43 ± 4.59	0.88^**†**^	**0.04**^**†**^
AL (mm)	29.06 ± 1.27	29.16 ± 1.22	29.01 ± 0.88	29.31 ± 1.50	0.68^**†**^	0.38^**†**^

Statistically significant *P*-values are shown in bold

*BCVA* best-corrected visual acuity, *SER* spherical equivalent refraction, *AL* axial length, *P1* the comparison of the NMTM and MTM groups, *P2* the comparison of the MTM Stage 1 and MTM Stage 2

^*^Chi-square test

^†^*t*-test

^‡^*U* test

### Optic disc region structure and microcirculatory parameters

Compared to that of the NMTM group, the DFD was longer (*P* < 0.019), optic disc tilt was greater (*P* < 0.001), PPA area was larger (*P* < 0.001), and PPA/ONH was higher (*P* < 0.001) in the MTM group. The PST was lower in the MTM group than in the NMTM group (*P* < 0.001) (Table 2). In addition, the mean PCT (*P* < 0.001) and PCT in the 10 orientations were significantly lower in the MTM group than in the NMTM group (all *P* < 0.01) (Table 3). Vascular density in the nanosuperior (NS) region of the optic disc was significantly lower in the MTM group (*P* = 0.037), and the temporoinferior (TI) and temporosuperior (TS) were significantly higher (all* P* < 0.05). MTM stage 2 had lower vascular density in the peri-optic disc region (2–4 mm) (*P* = 0.043) and SN region (*P* = 0.026) (Table 4).

**Table 2:** Comparison of optic disc morphology of the NMTM and MTM groups

Parameters	NMTM group	MTM group			P1	P2
	(*n* = 50)	Total (*n* = 50)	MTM Stage 1 (*n* = 25)	MTM Stage 2 (*n* = 25)		
Optic disc maximal diameter (mm)	1.78 ± 0.38	1.89 ± 0.49	1.93 ± 0.55	1.86 ± 0.44	0.213^**†**^	0.625^**†**^
Optic disc, minimal diameter (mm)	1.40 ± 0.43	1.21 ± 0.49	1.42 ± 0.52	1.18 ± 0.47	0.05^**†**^	0.681^**†**^
Optic disc tilt ratio	1.34 ± 0.31	1.69 ± 0.48	1.67 ± 0.51	1.71 ± 0.45	**0.000**^**‡**^	0.479^**‡**^
Optic disc rotation degree (°)	21.83 ± 14.96	20.31 ± 13.66	20.66 ± 15.72	19.96 ± 11.56	0.967^**‡**^	0.641^**‡**^
The area of optic disc (mm^2^)	2.20 ± 0.86	1.98 ± 1.01	1.95 ± 0.95	2.02 ± 1.08	0.156^**‡**^	0.892^**‡**^
DFD (mm)	5.83 ± 0.65	6.16 ± 0.74	6.12 ± 0.76	6.21 ± 0.73	**0.019**^**†**^	0.706^**†**^
PPA area (mm^2^)	3.20 ± 2.12	7.27 ± 4.79	6.21 ± 3.11	8.32 ± 5.91	**0.000**^**‡**^	0.121^**‡**^
PPA/ONH	1.49 ± 0.96	4.10 ± 2.75	3.67 ± 2.15	4.54 ± 3.24	**0.000**^**‡**^	0.271^**‡**^
PST (mm)	385.70 ± 52.16	305.30 ± 61.34	314.44 ± 49.17	296.16 ± 71.21	**0.000**^**†**^	0.297^**†**^

Statistically significant *P*-values are shown in bold

*DFD* optic disc-fovea distance, *PPA* peripapillary atrophy, *PPA/ONH* peripapillary atrophy/optic nerve head, *PST* peripapillary scleral thick, *P1* the comparison of the NMTM and MTM groups, *P2*: the comparison of the MTM Stage 1 and MTM Stage 2

^†^*t*-test

^‡^*U* test

**Table 3:** Comparison of peripapillary choroidal thickness of the NMTM and MTM groups

Parameters	NMTM group	MTM group			P1	P2
	(*n* = 50)	Total (*n* = 50)	MTM Stage 1 (*n* = 25)	MTM Stage 2 (*n* = 25)		
Mean PCT (mm)	137.32 ± 26.83	109.46 ± 22.08	109.47 ± 18.61	109.50 ± 25.48	**0.000**^**‡**^	0.992^**‡**^
PCT (S) (mm)	129.47 ± 27.86	101.00 ± 24.13	100.66 ± 21.72	101.34 ± 26.78	**0.000**^**†**^	0.921^**†**^
PCT (I) (mm)	139.61 ± 31.96	111.57 ± 25.83	110.85 ± 19.21	112.29 ± 31.50	**0.000**^**‡**^	0.839^**‡**^
PCT (NS) (mm)	147.57 ± 38.04	119.34 ± 32.20	116.81 ± 31.76	121.88 ± 33.09	**0.000**^**†**^	0.583^**†**^
PCT (NI) (mm)	148.80 ± 41.18	123.82 ± 36.95	120.74 ± 31.38	126.90 ± 42.23	**0.002**^**†**^	0.561^**†**^
PCT (IN) (mm)	147.46 ± 49.44	117.59 ± 36.12	114.89 ± 20.95	120.29 ± 47.01	**0.000**^**‡**^	0.808^**‡**^
PCT (IT) (mm)	130.92 ± 48.27	98.56 ± 32.38	105.82 ± 34.95	91.30 ± 28.46	**0.000**^**‡**^	0.109^**‡**^
PCT (TI) (mm)	124.30 ± 36.64	98.30 ± 31.47	97.25 ± 29.32	99.35 ± 34.06	**0.000**^**‡**^	0.900^**‡**^
PCT (TS) (mm)	123.78 ± 42.72	92.37 ± 28.51	95.06 ± 23.70	89.67 ± 32.91	**0.000**^**†**^	0.510^**†**^
PCT (ST) (mm)	118.28 ± 33.12	87.17 ± 25.92	86.47 ± 23.06	87.88 ± 28.97	**0.000**^**‡**^	0.884^**‡**^
PCT (SN) (mm)	135.78 ± 33.60	110.91 ± 37.30	108.64 ± 31.28	113.17 ± 43.04	**0.001**^**†**^	0.672^**†**^

Statistically significant *P*-values are shown in bold

*PCT* peripapillary choroidal thickness, *P1* the comparison of the NMTM and MTM groups, *P2* the comparison of the MTM Stage 1 and MTM Stage 2

^†^*t*-test

^‡^*U* test

**Table 4:** Comparison of optic disc and peri-optic disc vascular density of the NMTM and MTM groups

Parameters	NMTM group (*n* = 50)	MTM group			P1	P2
		Total (*n* = 50)	MTM Stage 1 (*n* = 25)	MTM Stage 2 (*n* = 25)		
VD (0–2 mm) (%)	63.37 ± 11.99	65.17 ± 11.06	68.19 ± 11.41	62.15 ± 10.03	0.438^**†**^	0.053^**†**^
VD (2–4 mm) (%)	59.85 ± 8.56	57.91 ± 7.56	60.06 ± 7.42	55.76 ± 7.20	0.232^**†**^	**0.043**^**†**^
VD (NS) (%)	45.24 ± 13.82	39.69 ± 13.07	42.11 ± 13.37	37.27 ± 12.57	**0.037**^**‡**^	0.133^**‡**^
VD (NI) (%)	42.43 ± 12.68	38.33 ± 14.43	38.58 ± 13.33	38.09 ± 15.73	0.135^**†**^	0.906^**†**^
VD (IN) (%)	57.31 ± 11.50	54.13 ± 13.30	55.19 ± 12.60	53.07 ± 14.14	0.204^**†**^	0.578^**†**^
VD (IT) (%)	71.65 ± 14.52	71.82 ± 12.01	73.44 ± 13.91	70.19 ± 9.76	0.715^**‡**^	0.190^**‡**^
VD (TI) (%)	74.05 ± 12.48	77.91 ± 14.72	80.36 ± 8.59	75.47 ± 18.88	**0.018**^**‡**^	0.648^**‡**^
VD (TS) (%)	76.72 ± 9.40	80.38 ± 10.51	81.93 ± 7.63	78.83 ± 12.74	**0.020**^**‡**^	0.455^**‡**^
VD (ST) (%)	71.26 ± 10.26	67.70 ± 14.35	71.25 ± 9.70	64.14 ± 17.33	0.356^**‡**^	0.256^**‡**^
VD (SN) (%)	54.23 ± 13.63	50.05 ± 15.10	54.75 ± 13.48	45.36 ± 15.42	0.150^**†**^	**0.026**^**†**^

Statistically significant *P*-values are shown in bold

*VD* vascular density, *P1* the comparison of the NMTM and MTM group, *P2* the comparison of the MTM Stage 1 and MTM Stage 2

^†^*t*-test

^‡^*U* test

### Parameters significantly correlated with MTM

Through the GEE we can explore the relationship between the MTM, optic disc structure, and microcirculatory parameters. PPA area (*B* =  − 0.436, *P* = 0.028), the mean PCT (*B* =  − 0.436, *P* = 0.028), PCT (S) (*B* = 0.145, *P* = 0.027), PCT (IN) (*B* = 0.128, *P* = 0.013), PCT (TI) (*B* = 0.078, *P* = 0.040), and PST (*B* = 0.015, *P* = 0.046) were correlated with MTM (Table 5).

**Table 5:** Analysis of correlations between MTM and variables by generalized estimating equation

Parameters	*B* value	Standard error	Wald value	OR	95% CI	*P*
Age (y)	− 0.012	0.029	0.167	0.988	0.934–1.046	0.683
BCVA (logMAR)	1.280	1.928	0.441	3.598	0.082–157.604	0.507
Optic disc tilt ratio	− 2.982	1.687	3.121	0.051	0.002–1.386	0.077
PPA area (mm^2^)	− 0.436	0.198	4.821	0.647	0.438–0.954	**0.028**
PPA/ONH	− 0.554	0.406	1.279	0.575	0.220–1.501	0.261
Mean PCT (mm)	− 0.646	0.242	7.112	0.524	0.326–0.843	**0.008**
PCT (S) (mm)	0.145	0.066	4.861	1.157	1.016–1.316	**0.027**
PCT (I) (mm)	0.087	0.075	1.328	1.091	0.941–1.264	0.249
PCT (NS) (mm)	0.087	0.048	3.258	1.091	0.993–1.200	0.071
PCT (NI) (mm)	0.008	0.036	0.046	1.008	0.939–1.082	0.830
PCT (IN) (mm)	0.128	0.051	6.158	1.137	1.027–1.258	**0.013**
PCT (IT) (mm)	0.032	0.025	1.633	1.033	0.983–1.085	0.201
PCT (TI) (mm)	0.078	0.038	4.198	1.081	1.003–1.166	**0.040**
PCT (TS) (mm)	0.032	0.028	1.257	1.032	0.977–1.091	0.262
PCT (ST) (mm)	0.067	0.044	2.282	1.070	0.980–1.167	0.131
PCT (SN) (mm)	0.018	0.026	0.440	1.018	0.966–1.072	0.507
VD (NS) (%)	− 0.037	0.029	1.552	0.964	0.910–1.021	0.213
VD (TI) (%)	0.003	0.053	0.002	1.003	0.903–1.113	0.961
VD (TS) (%)	− 0.012	0.057	0.041	0.988	0.884–1.106	0.839
PST (mm)	0.015	0.007	3.988	1.015	1.000–1.031	**0.046**
DFD (mm)	− 0.031	0.675	0.002	0.963	0.258–3.645	0.969

Statistically significant *P*-values are shown in bold

*B* unstandardized coefficient, *BCVA* best-corrected visual acuity, *PPA* peripapillary atrophy, *PPA/ONH* peripapillary atrophy/optic nerve head, *PCT* peripapillary choroidal thickness, *VD* vascular density, *PST* peripapillary scleral thick, *DFD* optic disc-fovea distance, *S* superior, *I* inferior, *NS* nasosuperior, *NI* nasoinferior, *IN* inferonasal, *IT* inferotemoporal, *TI* temporoinferior, *TS* temporosuperior, *ST* superotemporal, *SN* superonasal

### Linear correlation between PST and ocular parameters

**Fig. 3:**
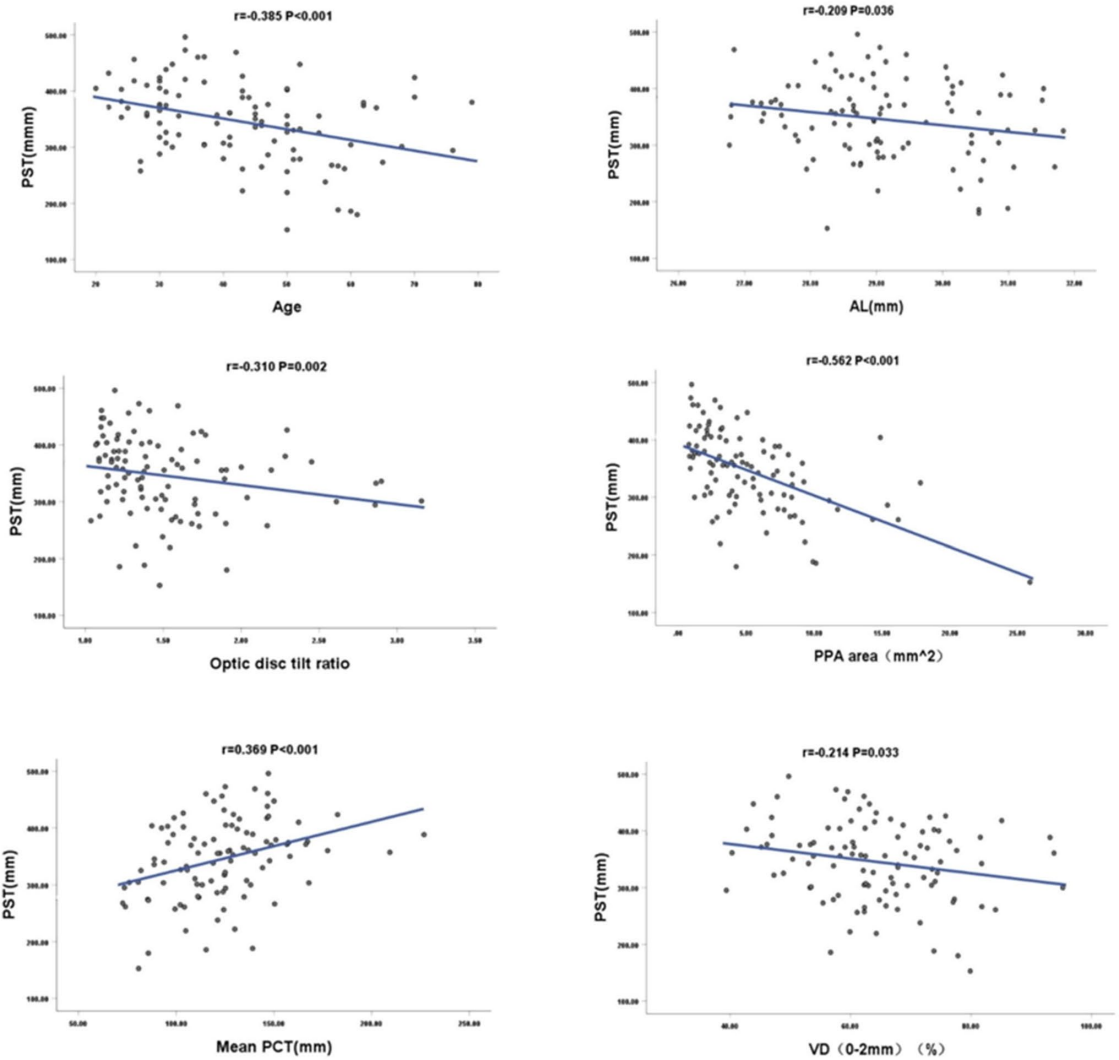
The correlation between PST and age, AL, optic disc tilt ratio, PPA area, mean PCT, and VD in the central region of the optic disc in 100 high myopic eyes were demonstrated. AL, axial length; PPA, peripapillary atrophy; PCT, peripapillary choroidal thickness; VD, vascular density

PST was significantly correlated with age (*r* =  − 0.385, *P* < 0.001), and the older the age, the thinner the PST. There was a significant correlation with AL (*r* =  − 0.209, *P* = 0.036); the longer the AL, the thinner the PST. There was a significant correlation with optic disc tilt (*r* =  − 0.310, *P* = 0.002); the greater the optic disc tilt, the thinner the PST. Further, there was a significant correlation with the PPA area (*r* =  − 0.562, *P* < 0.001); the larger the PPA area, the thinner the PST. There was a significant correlation with mean PCT (*r* = 0.369, *P* < 0.001); the thinner the mean PCT, the thinner the PST, and a significant correlation with vascular density in the central region of the optic disc (0–2 mm) (*r* =  − 0.214, *P* = 0.033); the greater the vascular density in the central region of the optic disc, the thinner the PST. There was no correlation between SE and peri-optic disc area (2–4 mm) vascular density (all *P* > 0.05) (Table 6)(Fig. 3).

**Table 6:** Linear correlation between PST and ocular parameters

Parameters	PST	
	*r* value	*p* value
Age (y)	− 0.385	** < 0.001**
SER (D)	0.069	0.498
AL (mm)	− 0.209	**0.036**
Optic disc tilt ratio	− 0.310	**0.002**
PPA area (mm^2^)	− 0.562	** < 0.001**
Mean PCT (mm)	0.369	** < 0.001**
VD (0 − 2 mm) (%)	− 0.214	**0.033**
VD (2 − 4 mm) (%)	0.082	0.418

Statistically significant *P*-values are shown in bold

*AL* axial length, *SER* spherical equivalent refraction, *PPA* peripapillary atrophy, *PCT* peripapillary choroidal thickness, *VD* vascular density

## Discussion

This study is the first to report the relationship between the structure and microcirculation of the optic disc region and MTM in the context of eye-axis matching. AL and PS were previously considered important factors affecting the optic disc structure and microcirculation [20]; however, our study found that MTM was also a risk factor for changes in the optic disc region. Since the sclera is an important factor in high myopic fundus degeneration, we further explored the factors associated with PST.

Patients in the MTM group were older than those in the NMTM group (47.52 ± 12.19 vs. 38.08 ± 13.00 years). This suggests that age may be a factor influencing the presence of MTM, which has been reported in previous studies [21]; however, some studies have shown that the main cause of reduced microcirculation in the optic disc region in people with high myopia is not age [20, 22]. The GEE did not show a correlation between age and MTM. Therefore, we believe that age difference had no significant effect on our analysis of the structure and microcirculation. 

The optic disc tilt in the MTM group was significantly greater, the DFD was significantly longer, and the PPA area and PPA/ONH ratio were significantly greater than those in the NMTM group; the GEE equation suggested that the PPA area was significantly correlated with MTM. This result is in agreement with previous studies [8]. PPA appears in the early stages of high myopia and is a characteristic change in the myopic fundus. With the development of high myopia, the Bruch’s membrane becomes actively elongated and thinned [23]. Atrophy of the retina and choroid gradually worsens, and with the emergence of MTM, the PPA area is further increased. To some extent, the PPA area increase can reflect levels of chorioretinal atrophy in the optic disc region [24], and a larger PPA area is related to an increase in optic disc tilt [6]. In addition to AL, MTM is a risk factor for PPA. PST was significantly lower in the MTM group than in the NMTM group; uneven atrophy of the choroid may be the reason for the morphological changes of the sclera [25], and the change of sclera morphology may lead to the abnormality of fundus morphology and microcirculation in the optic disc region. The tilt of the optic disc is due to the change in the angle of the optic nerve entering the scleral foramen, which is greater in eyes with MTM owing to the thinning of the PST and the change in morphology. The increase in the PPA area is associated with peripapillary scleral atrophy [26]; as the sclera thins, the degree of chorioretinal damage increases, and further loss of choroidal vessels and the PPA area increases. Therefore, we speculate that morphological changes in the fundus optic disc region occurred after the sclera became thinner.

The mean peripapillary choroidal thickness and PCT in 10 orientations in the MTM group were significantly lower; the GEE indicated that PCT was associated with MTM. PST thinning has also been observed in high myopic eyes [27]. We found that PCT continued to decrease with the advent of MTM, possibly due to the development of pathologic myopia. Oxygen consumption of the retina further increased, while the compensatory effect of the choroid further decreased, resulting in its thinning [28]. Zhang et al. [29] found that the choroid participates in signal transmission from the retina to the sclera and in the occurrence of scleral morphological changes. Therefore, the PCT level may be a predictor of MTM. Comparison of the intra- and peri-optic disc vascular density in the MTM and NMTM groups revealed that the vascular density increased in the optic disc (0–2 mm), TS, TI, and IT directions; the vascular density increased significantly in the TS and TI directions and decreased in the other directions. This tendency for increased vascular density is particularly evident in MTM stage 1 and decreased in MTM stage 2. Previous studies have shown that in high myopia, the temporal vascular density is higher, which is consistent with our findings. It may be due to the preferential perfusion of blood to the arcuate nerve fibre region located temporally during fundus deformation, thus preserving retinal perfusion in critical areas and ensuring normal visual function [20]. We speculate that with the emergence of MTM, temporal vascular density increases compensatively to maintain normal visual function. However, with the aggravation of MTM, the choroid atrophies further; this compensatory effect disappears, and the vascular density in and around the optic disc decreases. This explains why the reduction in BCVA was not significant at MTM stage 1, whereas it was significantly reduced at MTM stage 2. However, it is unclear whether the decrease in blood perfusion results from MTM or is involved in the occurrence of MTM.

PST correlated with both axial length and age in high myopia. The PST in high myopia becomes thinner with the growth of age and axial length. PST was negatively correlated with optic disc tilt, PPA area, and optic disc vascular density (0–2 mm) and positively correlated with mean PST. Previous studies have revealed that subfoveal scleral thickness is negatively correlated with age and axial length, and positively correlated with subfoveal choroidal thickness [30, 31]. We arrived at similar conclusions in the PST study. While PST was negatively correlated with optic disc vascular density (0–2 mm), it is possible that in the early stages of MTM, the sclera begins to become thinner, but the vascular density in the optic disc increases to maintain normal visual function. The shape and structure of the sclera change with an increase in the axial length, a process called scleral remodelling. Ohno-Matsui et al. [32] found that the posterior sclera of high myopia became thinner, especially due to the abnormal expansion of the sclera in the optic disc region, which led to eyeball deformation. We speculate that the emergence and progression of MTM may lead to abnormal microcirculation in the optic disc. The thinning choroid leads to scleral remodelling due to an insufficient supply of oxygen. When the sclera becomes very thin, it can no longer maintain its normal shape and cannot protect the intra- and peri-disc tissues, disrupting the optic disc structure. The scleral tissue and biomechanics play important roles in the occurrence of MTM. However, there are no studies on whether changes in scleral thickness accelerate the occurrence of pathological myopia.

The present study has several limitations. First, case–control studies do not truly reflect the effects of changes over time, and it is unclear whether changes in optic disc structure and microcirculation in high myopia occur before or after morphological changes; further cohort studies are needed. Second, because of the poor quality of OCT/OCTA imaging in patients with severe MTM, only patients with stage 1 and 2 MTM were selected, and not all patients with stage MTM could be studied. Third, even if the axial lengths match, all confounding factors of the MTM cannot be completely excluded. Further research is required to expand the sample size in this study.

## Conclusion

MTM was associated with abnormalities in optic disc structure and microcirculation, and the PPA area, PST, and PCT were significantly associated with MTM. PST thinning was associated with PCT and microcirculation. Understanding the morphological features and factors influencing the optic disc region may provide insight into the pathogenesis of MTM and the development of pathologic myopia.

## Data Availability

Data are available upon request.
